# Improving Likert scale big data analysis in psychometric health economics: reliability of the new compositional data approach

**DOI:** 10.1186/s40708-024-00232-z

**Published:** 2024-07-10

**Authors:** René Lehmann, Bodo Vogt

**Affiliations:** 1 ifes Institute of Empiricism and Statistics, FOM University of Applied Science, Essen, 45127 Germany; 2 Chair in Business Administration, esp. in Empirical Economics, and Health Economics, Otto von Guericke University, Universitätsplatz 2, Magdeburg, 39106 Germany

**Keywords:** Bipolar Likert scale, Compositional data, Ilr transformation, Big data, Heavy-tailed distributions

## Abstract

Bipolar psychometric scales data are widely used in psychologic healthcare. Adequate psychological profiling benefits patients and saves time and costs. Grant funding depends on the quality of psychotherapeutic measures. Bipolar Likert scales yield compositional data because any order of magnitude of agreement towards an item assertion implies a complementary order of magnitude of disagreement. Using an isometric log-ratio (ilr) transformation the bivariate information can be transformed towards the real valued interval scale yielding unbiased statistical results increasing the statistical power of the Pearson correlation significance test if the Central Limit Theorem (CLT) of statistics is satisfied. In practice, however, the applicability of the CLT depends on the number of summands (i.e., the number of items) and the variance of the data generating process (DGP) of the ilr transformed data. Via simulation we provide evidence that the ilr approach also works satisfactory if the CLT is violated. That is, the ilr approach is robust towards extremely large or infinite variances of the underlying DGP increasing the statistical power of the correlation test. The study generalizes former results pointing out the universality and reliability of the ilr approach in psychometric big data analysis affecting psychometric health economics, patient welfare, grant funding, economic decision making and profits.

## Introduction

Psychologic big data is used for validating predictive models by applying a model developed on one dataset to a separate set of data or hold-out sample [1]. Concerning health economics, the statistical analysis of individual psychometric data and big data sets contributes to the derivation of standards and the evaluation of success of psychotherapeutic measures, e.g., via individual psychometric profiling and machine learning algorithms [2, 3]. Psychotherapeutic treatment and behaviour prediction both depend on the correct specification of personality facets and attitudes of individuals. Correct unbiased psychometric profiling can support the selection of apposite healthcare measures, reduce the costs of a treatment and save time. Moreover, the increase of patient welfare contributes to medical ethics.

Bipolar Likert scales (LS) are commonly used in psychology and medical psychometrics to establish norms and create psychological profiles of patients [4, 5]. Ensuring scientific rigor, it is crucial to have a thorough understanding of the relationships and impacts between variables, as well as the effectiveness of therapeutic interventions [6]. The success of treatment and its outcomes rely on accurate standards and the patient’s psychological profile. Inadequate data analysis can lead to biased standards, which can in turn distort machine learning algorithms, impacting psychological profiling and medical diagnostics. This can result in false positive or negative diagnoses for medical borderline cases. Moreover, flawed psychological profiling may contribute to misdiagnoses, compromised treatment plans, increased healthcare costs, and ultimately harm patient well-being. Therefore, it is essential to employ unbiased statistical methods that offer high statistical power [7].

Recently, [8] uncovered the compositional structure of bipolar scales data. As discussed by [9–12], analyzing compositional data is complex due to the underlying Aitchison metric. The compositional data space, known as the Simplex, is inherently non-linear, making traditional measures of linear association like the Pearson correlation coefficient or linear regression techniques unsuitable [9, 13, 14]. Linear regression methods such as moderator and mediator analyses that rely on (partial) correlations can be biased [15]. In psychometric big data analytics, the focus is on structure and correlation rather than causation, such as exploring the relationship between psychological data and workplace risk [16]. However, big data technology can produce spurious correlations [17]. Consequently, psychological assessments based on correlation-based approaches like partial least squares structural equation modeling (PLS SEM) [18] may also be suboptimal, leading to increased costs and less effective healthcare interventions.

Neglecting the Simplex introduces bias into statistical analysis, such as in statistical hypothesis testing or in the estimation of psychometric standards [10, 19]. Highlighting the inherent bias in measures of association like the Pearson correlation, [8] proposed the isometric log-ratio (ilr) transformation, which yields interval-scaled real-valued data and unbiased results. Assuming a normally distributed data generating process (DGP), [8] and [14] present evidence that the ilr approach enhances the statistical power of well-known tests like correlation tests and paired and unpaired two-sample t-tests based on Student’s t-distribution.

Individual psychometric values are commonly expressed as the means or sums of item responses on a bipolar LS [20]. The central limit theorem (CLT) in statistics, with its various versions that accommodate non-i.i.d. random variables and other generalizations [21], ensures that the means and sums of ilr-transformed item response values are asymptotically normally distributed [22]. One of the key assumptions of the CLT is that the variance contributions of the individual components are small.

When dealing with big data sets, it is reasonable to consider the existence of extreme values and high variance, which could potentially undermine the applicability of the CLT. For instance, a heavy-tailed DGP may slow down the convergence of means and sums towards a normally distributed random variable. Additionally, a DGP with infinite variance makes the CLT infeasible. While most standard statistical methods are resilient to deviations from assumptions like normality in data distribution [23, 24], exploring the ilr approach under such extreme conditions is highly valuable.

Consider the correlation test of the null-hypothesis $H_0:\ \varrho =0$ using Student’s t-distribution ([25]) where $\varrho$ denotes the true coefficient of correlation. Via simulation we provide evidence that the ilr approach yields satisfactory results if the CLT is violated. Contrasted with conventional analyses, the statistical power of the popular correlation test relying on Student’s t-distribution improves when the DGP exhibits heavy-tailed characteristics or infinite variance. In other words, the ilr approach performs well under extreme conditions, leading to more dependable data-driven decisions. Consequently, there is potential to lower collection costs while preserving or even enhancing statistical power compared to traditional statistical data analysis.

## Literature review

As noted by [8, 12, 26] compositional data structures in psychometric measure scales can be overseen, e.g., regarding Thurstonian scales and bipolar LS. Thurstonian scales offer test persons a set of alternatives. A participant allocates percentages or absolute scores to the different alternatives. Simplex data can also be found in statistical geology where data points represent the compositions of concentrations of chemical elements in different soil samples [15, 19, 27]. Compositional data also appear in economics. For example, consider a company value split into its contributing parts (value of the machine park, value of assets, value of property assets etc.) or consider the contributions to the gross domestic product of different countries.

There has been much effort in providing adequate statistical approaches to analyze Simplex data, among them the logit transformation, the additive log-ratio (Alr) and the centered log-ratio (Clr) transformation. Later, the ilr transformation was introduced [9, 27]. The approaches have advantages and disadvantages. Let $x=(x_1,\ldots ,x_D)\in \mathbb R^D$ be a compositional data point according to section 3.2. That is, $x_i>0\ \forall i=1,\ldots ,D$ and $\sum _{i=1}^D{x_i}=\kappa$ for some $\kappa \in \mathbb R$. From $\sum _{i=1}^D{x_i}=\kappa$ it follows that any $x_i$ (e.g., $x_1$) of the composition can be deleted without losing information. For example, the deleted value $x_1$ is obtained via $x_1=\kappa -\sum _{i=2}^D{x_i}$. That is, the composition contains a redundancy affecting statistical analysis. The alr aims to eliminate an arbitrary redundant value, say $x_j$. It is defined as

1 $$\begin{aligned} alr((x_1,\ldots ,x_D)^T)=\left( \ln \frac{x_1}{x_j},\ldots ,\ln \frac{x_{j-1}}{x_j},\ln \frac{x_{j+1}}{x_j},\ldots \ln \frac{x_D}{x_j}\right) ^T \end{aligned}$$

where $j\in \{1,\ldots ,D\}$ is arbitrarily chosen. The alr is subjective because the results depend on the choice of *j*. However, if $D=2$ the choice is not subjective and the alr reduces to the logit transformation

2 $$\begin{aligned} logit((x_1,x_2)^T)=alr((x_1,x_2)^T)=\ln \frac{x_1}{x_2}=\ln \frac{x_1}{\kappa -x_1}. \end{aligned}$$

Choosing the geometric mean as the denominator of all components the clr avoids the subjectivity of the alr.

3 $$\begin{aligned} clr((x_1,\ldots ,x_D)^T)=\left( \ln \frac{x_1}{\left( \prod _{i=1}^D{x_i}\right) ^{1/D}},\ldots ,\ln \frac{x_D}{\left( \prod _{i=1}^D{x_i}\right) ^{1/D}}\right) ^T \end{aligned}$$

Please note that the number of components of the clr transformed data point equals *D*. If $D=2$ the clr reduces to

4 $$\begin{aligned} clr((x_1,\kappa -x_1)^T)=\left( \ln \frac{x_1}{\sqrt{x_1(\kappa -x_1)}},\ln \frac{\kappa -x_1}{\sqrt{x_1(\kappa -x_1)}}\right) ^T=\left( 0.5\ln \frac{x_1}{\kappa -x_1},0.5\ln \frac{\kappa -x_1}{x_1}\right) ^T. \end{aligned}$$

That is, the first component of the clr differs from the alr and the logit by the factor 0.5. Obviously, the first and the second component of the clr are related via $0.5\ln \frac{\kappa -x_1}{x_1}=0.5(\ln (\kappa -x_1)-\ln (x_1))=-0.5(\ln (x_1)-\ln (\kappa -x_1))=-0.5\ln \frac{x_1}{\kappa -x_1}$. It can be summarized that the clr does not eliminate a redundancy but it avoids the subjectivity of the alr. The alr eliminates a redundancy but the arbitrary choice of $x_j$ affects subsequent statistical analysis. If $D=2$, however, the alr is not subjective and equals the logit. In this paper we propose the ilr transformation because it avoids redundancies and subjectivity. For further details please refer to Sect. 3.2.

Simplex data must not be evaluated using methods designed for interval data [9]. For example, Pearson correlations *r* are biased estimates of the true correlation $\varrho$ if the compositional structure is ignored [19]. The accurate measurement of criterion-related validity is essential for ensuring the quality of psychometric evaluations. Inaccuracies in measuring mean values and standard deviations (as discussed in [10]) can lead to biased psychometric standards, thereby compromising psychotherapeutic assessments and managerial decision-making.

These limitations also impact statistical power. As highlighted by [28–30], the issue of low statistical power (“underpowerment”) and results hovering near the threshold of significance should not be overlooked in psychometric analyses. Lehmann and Vogt [31, 32] present findings indicating that the ilr approach induces a movement towards normality. This means that the alignment of means and sums of item response values with a normally distributed random variable is enhanced, thereby influencing the statistical power of methods reliant on approximately normally distributed data.

Compositional data should not be evaluated using standard statistical procedures. Evaluation of the ilr-transformed data instead of the raw data is expedient [33, 34]. Finally, the results can be back-transformed by means of the inverse ilr transformation [8, 11].

## Materials and methods

This section provides a brief overview of the ilr approach and related psychometric parameters (e.g., the limit of quantification (LOQ)). The simulation process is described including different DGP and other simulation parameters.

For proper understanding of the different types of scales, it is necessary to distinguish between statements (i.e., items of a questionnaire) and their corresponding response scale (RS) as well as a LS (i.e., a set of items represented by the sum or mean value of their corresponding responses) and the scale of interest (SOI, e.g., a continuous scale of all possible manifestations of a trait). The RS measures the order of magnitude of a person’s agreement (OMA) or disagreement (OMD) towards a statement. Associating verbal responses (e.g., ranging from “not at all” to “very much”) with numerical values (e.g., $1,\ldots ,5$) is common practice [35, 36]. The LS represents a model of the SOI for estimating the order of magnitude of a personality trait or attitude (OMT) [20]. In the following, if not otherwise stated, the term scale refers to a bipolar scale and the term construct refers to a psychological construct.

### Bipolar constructs and psychometric scales

Psychometric scales provide estimates of individual values of constructs. For example, think of the Big 5 trait openness. The items of a questionnaire (e.g., the BFI-10 inventory of [37]) cover specific aspects of a construct. Considering an overall value of the item responses (e.g., the arithmetic mean) provides an individual estimate of the OMT.

**Fig. 1:**
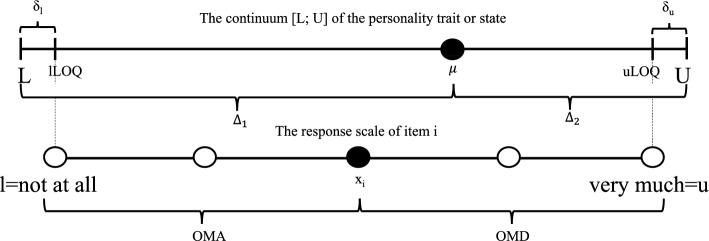
Illustration of the different types of scales used in psychometrics. The continuum [L; U] represents the TS. The lower scale represents the RS

Due to imperfect knowledge, uncertainty about situations and a complex environment [38–40] the psychometric scale cannot cover all individual manifestations of the construct implying the existence of a LOQ [8]. For an illustration see Fig. 1.

The continuum [L; U] contains all possible individual manifestations of a construct ranging from a minimum value L (e.g., non-openness to anything) to a maximum value U (e.g., openness to everything). A person’s order of magnitude of the construct (say, $\mu$) is located within these bounds. Moreover, the complements $\Delta _1$ and $\Delta _2$, both represent the order of magnitude of the construct. We have $\Delta _1+\Delta _2=U-L$. For example, set L=0, U=100, $\mu =70$, $\Delta _1=70$ and $\Delta _2=30$.

The psychometric scale comprises various items indexed as $i=1,\ldots ,I$, each linked to a response scale that spans from “not at all” to “very much”, denoted as lower (l) and upper (u) limits. Since the items may not encompass all facets of the construct, the lower and upper limits of the response scale differ from L and U, representing the lower (lLOQ) and upper (ULOQ) limits of quantification. The unaddressed region at the boundaries of the construct scale, not accounted for by the items and their corresponding response scale, is referred to as $\delta _l$ and $\delta _u$.

Any response $x_i$ towards an item assertion reflects the OMA and the OMD towards the item assertion. For example, let l=lLOQ=2.5, u=uLOQ=97.5, $\mu =60$ $x_i=50=OMA$, $OMD=50$, $\delta _l=[0;2.5)$ and $\delta _u=(97.5;100]$). That is, $x_i=50$ estimates the unknown value of $\mu =60$ and the pair $(50,50)^T$ denotes a so-called (bivariate) compositional data point.

### The compositional structure in brief

According to [8] $L<U$ can be chosen arbitrarily. In the following set $L=0$ and $U=100$. Without loss of generality consider a RS $r=\{r_1,\ldots ,r_{k+1}\}$ with $r_1=1$, $r_{k+1}=k+1$, $k\in \mathbb N$, $r_{s+1}-r_s=1$ $\forall s\in {1,\ldots ,k}$ (e.g., the discrete scale $\{1,2,3,4,5\}$ of $k+1=5$ categories ranging from “not at all (1)” to “very much (5)”).

Let $p\in (0;1)$ quantify the LOQ. Symmetric values of lLOQ and uLOQ are assumed, that is, $lLOQ=100\cdot p/2$ and $uLOQ=100(1-p/2)$ [12, 31]. Therefore, the unaddressed regions at the boundaries are also symmetric with $\left| \delta _l\right| =\left| \delta _u\right| =p/2$. Let $x'\in \{r_1,\ldots ,r_{k+1}\}$ be an observed response value. The 4-step-algorithm presented below transforms any response value $x'$ towards the trait scale [0; 100] with due regard to *p*.

1. Choose $p\in (0;1)$. Set $lLOQ=100\cdot p/2$ and $uLOQ=100\cdot (1-p/2)$ (e.g., $p=0.05$, lLOQ=2.5, uLOQ=97.5)
2. Define the $range:=uLOQ-lLOQ$ and the step width $sw:=range/k$ (e.g., $range=97.5-2.5=95$ and $sw=95/4=23.75$).
3. Let the observed response value be $x'=r_s\in \{r_1,\ldots ,r_{k+1}\}$ with $s\in \{1,\ldots ,k+1\}$ (e.g., $x'=3$ corresponds to $s=3$).
4. Calculate the response value $x^*=lLOQ+sw\cdot (s-1)$ (e.g., $x'=3$ and $x^*=2.5+23.75\cdot (3-1)=50$).

For example, the algorithm transforms the RS $r=\{1,2,3,4,5\}$ towards the RS* $r^*=\{2.5,26.25,50,73.75,97.5\}$ ($p=0.05$). Please note that the bounds of $r^*$ depend on *p*. $x^*\in (lLOQ; uLOQ)$ reflects the transformed OMA towards the item assertion. Any OMA value implies a complementary OMD value, say $100-x^*$. Define $x=(x_1,x_2)^T\in \mathbb R^2$ with $x_1:=x^*$, $x_2:=100-x^*$, $x_1,x_2>0$ and $x_1+x_2=100$.

**Fig. 2:**
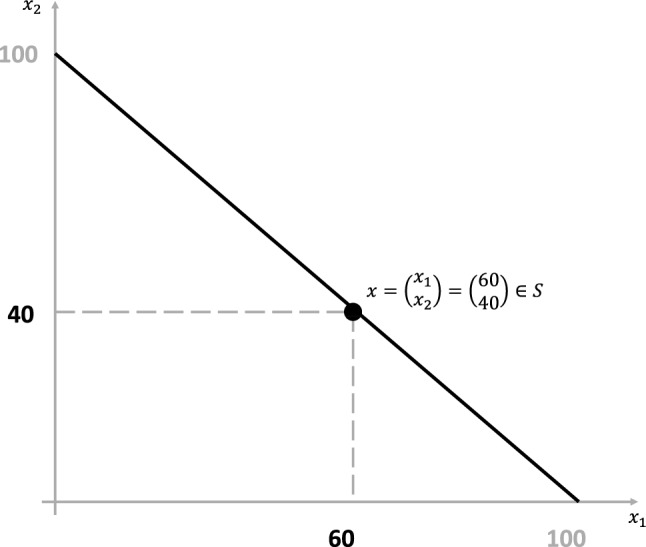
The black line illustrates the Simplex of bipolar scales data. $x_1$ ($x_2$) represents the OMA (OMD) towards the item assertion, respectively. The exemplary point $x=(60,40)^T$ illustrates an OMA of 60 and an OMD of 40

Generally, the compositional data space is defined as ${\mathcal{S}}: = \left\{ {x = (x_{1} , \ldots ,x_{D} )^{T}  \in {\mathbb{R}}^{D} |\sum\nolimits_{{i = 1}}^{D} {x_{i} }  = \kappa  \in {\mathbb{R}},x_{i}  > 0\forall i = 1, \ldots ,D} \right\}$. With $D=2$ and $\kappa =100$ the vector *x* fulfills the definition of compositional data [8, 27, 41, 42]. An illustration of the Simplex of bipolar scales data is presented in Fig. 2

### Ilr and inverse ilr transformation

Any compositional data point *x* depends on the Aitchison metric [10]. However, most standard statistical procedures (e.g., computation of arithmetic means, Pearson correlation, (multiple) linear regression, t-tests) are based on the Euclidean metric. The ilr transformation yields interval scaled data underlying the Euclidean metric [43]. By means of the ilr and the inverse ilr, data and statistical results (e.g., mean values) can easily be (back-)transformed. The ilr transformation is defined as $ilr(x)=ilr((x_1,\ldots ,x_D)^T):=(z_1,\ldots ,z_{D-1})^T$ with

5 $$\begin{aligned} z_s=\sqrt{\frac{s}{s+1}}\ln \frac{\root s \of {\prod _{j=1}^s{x_j}}}{x_{s+1}},\ s=1,\ldots ,D-1 \end{aligned}$$

In the present case of $D=2$ the ilr reduces to $ilr((x^*,100-x^*)^T)=z_1$ with

6 $$\begin{aligned} z_1=\sqrt{0.5}\ln \frac{x^*}{100-x^*}. \end{aligned}$$

For example, the ilr transform of the RS $r^*=\{2.5,26.25,50,73.75,97.5\}$ denotes $ilr((2.5,97.5)^T)=-2.59$, $ilr((26.25,73.75)^T)=-0.73$, $ilr((50,50)^T)=0$, $ilr((73.75,26.25)^T)=0.73$ and $ilr((97.5,2.5)^T)=2.59$. Please note that the bounds of the ilr RS depend on *p* because the bounds of $r^*$ depend on *p*. The smaller $p\in (0,1)$ is, the closer are the bounds of $r^*$ to 0 and 100, respectively. Therefore, $\lim \limits _{p\rightarrow 0}\frac{r_1^*}{r_{k+1}^*}=0$, $\lim \limits _{p\rightarrow 0}\frac{r_{k+1}^*}{r_1^*}=\infty$ and $\lim \limits _{p\rightarrow 0}\ln {\frac{r_1^*}{r_{k+1}^*}}=-\infty$, $\lim \limits _{p\rightarrow 0}\ln {\frac{r_{k+1}^*}{r_1^*}}=\infty$, i.e., the spread of the ilr RS increases as $p\rightarrow 0$.

The Simplex representation of the data can be obtained via the inverse ilr. It back-transforms any $z\in \mathbb R^{D-1}$ to an $x\in \mathcal S$. The inverse ilr is defined as follows. Let $z=(z_1,\ldots ,z_{D-1})^T\in \mathbb R^{D-1}$.

7 $$\begin{aligned} y_s&:=\sum _{j=s}^D{\frac{z_j}{\sqrt{j(j+1)}}}-\sqrt{\frac{s-1}{s}}z_{s-1};\ z_0:=z_D:=0 \end{aligned}$$

8 $$\begin{aligned} x_s&:=\kappa \cdot \frac{e^{y_s}}{e^{y_1}+\ldots +e^{y_D}},\ s=1\ldots ,D \end{aligned}$$

Like the ilr, the inverse ilr simplifies in the present case. The corresponding $x^*$ is obtained by setting $z_0:=z_D:=0$ and $\kappa =100$ with

9 $$\begin{aligned} x^*=100\cdot \frac{e^{y_1}}{e^{y_1}+e^{y_2}}\ \text {with}\ y_1=\sqrt{0.5}z_1\ \text {and}\ y_2=-\sqrt{0.5}z_1. \end{aligned}$$

Again, $x=(x^*,100-x^*)^T$ denotes the complete compositional data point. Applying the inverse ilr transformation to the ilr RS yields the RS $r^*$, e.g., invilr(0.73)=73.75 in the above example.

Please note that the simplified ilr transformation differs from the alr and the logit transformation only by the scaling factor $\sqrt{0.5}$, see section 2. The three transformations consider $\ln \frac{x^*}{100-x^*}$ in order to obtain interval scaled data. That is, mathematically they are practically identical if $D=2$. The idea of data evaluation is straight forward:

1. Apply the ilr transformation to obtain interval-scaled data.
2. Analyse the ilr transformed data using any appropriate statistical procedure (e.g., Shapiro-Wilk test, t-test, linear regression, Pearson correlation etc.)
3. Interpret the results on the interval scale.
4. If necessary: use the inverse ilr transformation to back-transform the results to the Simplex (e.g., apply the invilr to the arithmetic mean of ilr transformed data) and interpret.

### Simulation study on correlations

Correlations are often used to assess (e.g., criterion-related) validity or to quantify the order of magnitude of the linear association of variables (e.g. psychometric constructs). Furthermore, correlations contribute to the slope parameters of a linear regression model.

#### Implementation and parameters of the simulation

**Fig. 3:**
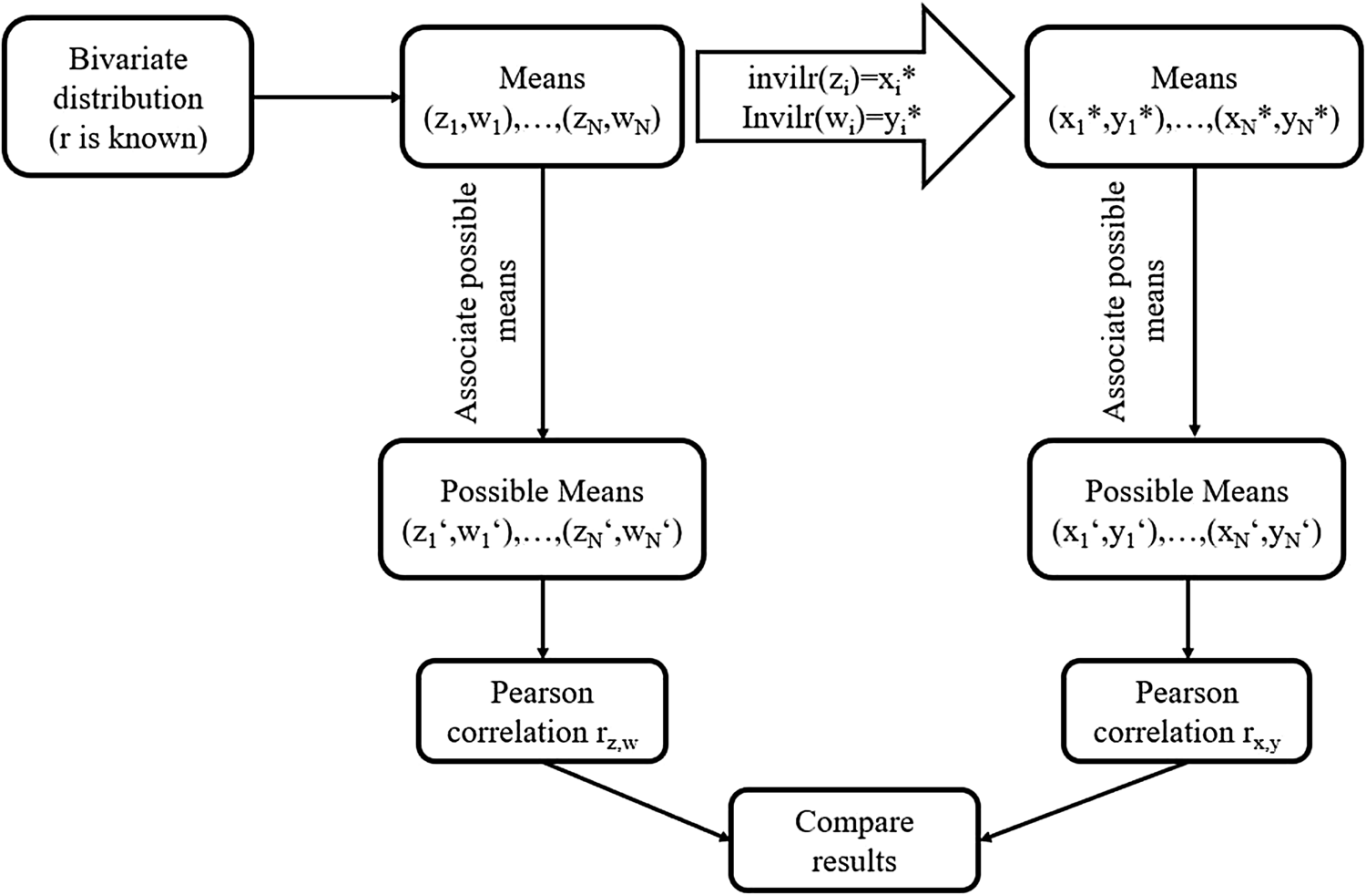
After simulating values using a bivariate distribution data are associated with their closest possible means (left-hand path). By means of the inverse ilr transformation the simulated values are transformed to the RS $r^*$ and associated with their closest possible means on the original RS (right-hand path). $H_0:\ \varrho =0$ is tested in both paths and the proportions of rejections of $H_0$ are obtained

Imagine two hypothetical personality traits, $T_1$ and $T_2$ (e.g., $T_1$=openness and $T_2$=risk disposition). Let $\zeta _1$ and $\zeta _2$ be a test individual’s order of magnitude of $T_1$ and $T_2$ in the ilr-transformed space. Let $z_1$ and $z_2$ be the means of the ilr-transformed item responses, that is, $z_i$ estimates $\zeta _i$ $(i=1,2)$. Using a bivariate distribution of a random vector $(Z_1,Z_2)^T$ with expectation $\mu \in \mathbb R^2$ and covariance matrix $\Sigma$ we simulate $(z_1,z_2)$ pairs. The simulation uses two DGP. First, a bivariate Laplace distribution is applied using the rmvl() function of the R package LaplacesDemons (for details refer to [44, 45]). Second, the bivariate Cauchy distribution is applied (see [46]) using the rmvc() function of the R package LaplacesDemons. For an illustration of the simulation procedure, see Fig. 3.

Please note that the first- and second-order moments of the Cauchy distribution do not exist. Thus, $\mu$ does not represent the expectation but the centre of the distribution. Additionally, $\Sigma$ denotes a positive definite scale matrix where $s_{11},\ s_{22}$ and $s_{12}$ refer to the terms “dispersion” and “codispersion”. The missing of first- and second-order moments also implies missing correlation $\varrho$. However, analogous to the bivariate Laplace distribution and the Pearson correlation, a measure of association *r* can be defined for two Cauchy distributed random variables (see [47]). Further details concerning different measures of association applicable to bivariate Cauchy distributions are presented by [48]. In the following, to provide a better reading of the text, the terms “dispersion”, “codispersion”, “centre of the distribution” and “measure of association” are replaced by the terms “variance”, “covariance”, “expectation” and “correlation”.

Without loss of generality, let us choose $\mu =(0,0)^T$ as the correlation $\varrho$ is independent of the distribution’s expectation but relies on the covariance $s_{12}$ and the variances $s_{11}$ and $s_{22}$ of $Z_1$ and $Z_2$, given by $\varrho =s_{12}/\sqrt{s_{11}s_{22}}$. By setting $s_{12}=\varrho \sqrt{s_{11}s_{22}}$ in the covariance matrix $\Sigma$, various correlations $\varrho \in {-0.65,-0.60,-0.55,\ldots ,0.55,0.6,0.65}\backslash {0}$ are examined. Previous research has shown that correlations with $\left| \varrho \right| >0.65$ do not need to be considered, as the findings consistently demonstrate 100% power regardless of analyzing data on the original scale or the ilr-transformed scale [8, 12].

To consider the influence of the variances on the statistical power, different values $s_{11},s_{22}\in \{0.25,0.5,\ldots ,1.75,2\}$ are used. Define $s^2=s_{11}+s_{22}$ as the total variance representing the overall dispersion.

Different values of $p\in \{0.02,0.04,\ldots ,0.2\}$ demonstrate the impact of the LOQ on statistical power. Various values of $k+1$, such as 5, 6, *and*10, are selected because the number of responses of the RS can influence the precision of trait measurement. Furthermore, the number of items in a scale (*I*) also plays a role in measurement accuracy. Hence, different values of $I\in \{1,4,10,30\}$ are employed for this purpose.

From the studies of [8, 12, 31, 32] it is known the the number of responses can have minor effects as well as the number of items. Also the underlying variances and the limit of quantification can affect the results. However, these studies were based on the assumption of a normally distributed DGP. For comparability we choose the same parameter ranges as proposed by [8].

Moreover, the parameter ranges seem reasonable. For example, the different values of *p* reflect measurement instruments of high (*p* close to 0), medium (*p* close to 0.1), and low (*p* close to 0.2) quality. A classic example in this context is measurement instruments for assessing the Big 5 personality traits. While the BFI-10 consists of 2 items per trait the NEO-FFI provides 12 items per trait. Assessing a person’s personality using two validated items cannot provide the quality of a measurement conducted using 12 validated items.

Societies can be more or less liberal, open minded etc. and the range of the manifestations of a trait in the population can vary between populations. For example, openness or diversity competence vary between intolerant and liberal societies implying smaller or larger variance of the orders of magnitude of a trait or state. Moreover, the variance also depends on the underlying population, that is, what we define as the population (e.g., one country vs. a union of countries vs. a continent). Thus, the range of possible construct values can vary suggesting larger or smaller variance of the DGP.

It is well-known that the number of responses of a response scale $\{1,\ldots ,k\}$ $(k\in \mathbb N)$ does not affect the validity of a psychometric scale [49] but increasing *k* can enhance the reliability of the measurements [50]. According to [14, 31] the number of responses *k* of the response scale $\{1,\ldots ,k\}$ can affect the results of the statistical analyses. Controlling for possible effects, we chose the common values.

Overall, the simulation incorporates a total number of $36\left( {\# {\text{variance}}\,{\text{combinations}}} \right) \cdot 26\left( {\# {\text{correlations}}} \right) \cdot 3\left( {\# k} \right) \cdot 4\left( {\# I} \right) \cdot 10\left( {\# p} \right) \cdot 2\left( {\# \,{\text{of}}\,{\text{DGP}}} \right) = 224,640$ scenarios. Each scenario is simulated 1000 times with 200 simulated pairs of means $(z_1,z_2)$, each. The statistical power of the correlation test of $H_0:\ \varrho =0$ in a specific scenario is given by the proportion of rejected null-hypotheses in 1000 simulation runs.

#### Associating simulated data to possible data

Calculating means of a finite number of item responses yields a discrete set of possible means. For example, using $I=2$ items and the ilr RS $r_1=-2.59,r_2=-0.73,r_3=0,r_4=0.73,r_5=2.59$ the set of possible means denotes $\{-2.59,-1.66,-1.30,-0.93,-0.73,-0.37,0,$ 0.37, 0.73, 0.93,  $1.30,1.66,2.59\}$. To obtain realistic values, any simulated mean $z_i$ $(i=1,2)$ is replaced with its nearest possible mean $\mu ^{ilr}_i$ ($i=1,2$) according to the Euclidean metric. In the above example the nearest possible mean of $z=0.82$ is given by $\mu ^{ilr}=0.93$. Note that the number of possible means depends on the number of responses $k+1$ and the number of items $I\in \mathbb N$.

The inverse ilr is used to transform any simulated random value towards the RS $r^*$. Replacing the inverse ilr tranformed value with its nearest possible mean yields a possible value. Although the Aitchison metric should be used on the RS $r^*$, the Euclidean metric is used to obtain the nearest possible mean. This approach is necessary because in common practice means and correlations are calculated without considering the compositional structure of the response data. The intention of the simulation is to show the effects of disregarding the compositional structure on the statistical analysis. Note that each possible mean of the RS $r^*$ corresponds to a possible mean of the original RS. Thus, any simulated mean $z_i$ $(i=1,2)$ could also be assigned to its nearest possible mean $\mu ^{orig}_i$ $(i=1,2)$ of the original RS. For example, let $z=0.82$ be a simulated mean and invilr(0.82)=76.13. Consider the RS $r=(1,2,3,4,5)$ and the RS $r^*=(2.5,26.25,50,73.75,97.5)$ consisting of $I=2$ items. The possible means of RS *r* are $\{0.5\cdot m\ |\ m=2,\ldots ,10\}$ while the possible means of RS $r^*$ are $\{2.5,14.38,26.25,38.13,50,61.88,73.75,85.63,97.5\}$. The nearest possible mean of 76.13 on the RS $r^*$ is given by 73.75 which represents the mean $\mu ^{orig}=4$ on the initial RS.

Each simulation run generates two data sets: $ILR=\{(\mu ^{ilr}_{1,i},\mu ^{ilr}_{2,i})\ |\ i=1,\ldots ,200\}$ and $ORIG=\{(\mu ^{orig}_{1,i},\mu ^{orig}_{2,i})\ |\ i=1,\ldots ,200\}$ and the correlation test based on Student’s t-distribution is applied to test $H_0:\ \varrho =0$ for the ILR and ORIG data sets. The two proportions of rejections of $H_0$ in 1000 runs represent the estimates of the statistical powers of the correlation test on both scales, the ilr scale and the original scale, that is, $Power^{ilr}$ and $Power^{orig}$. The difference $\Delta \ Power=Power^{ilr}-Power^{orig}$ indicates the superiority or inferiority of the ilr approach.

## Results of the simulation study and conclusions

**Fig. 4:**
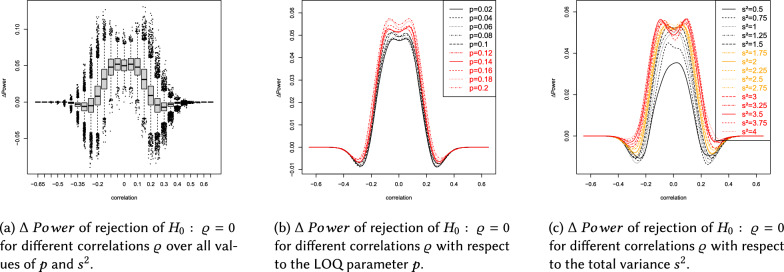
The data generating process is Laplace

**Fig. 5:**
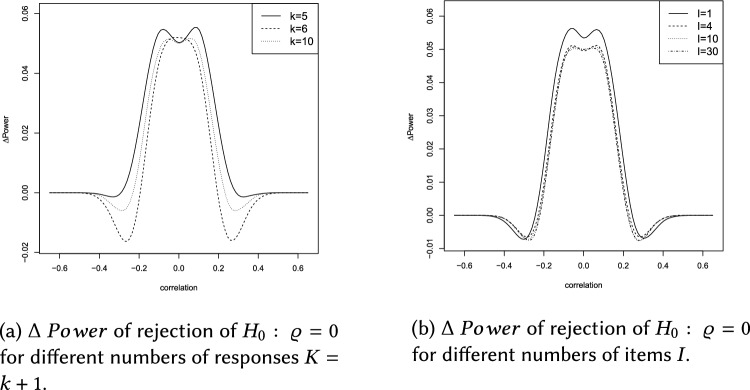
The data generating process is Laplace

**Fig. 6:**
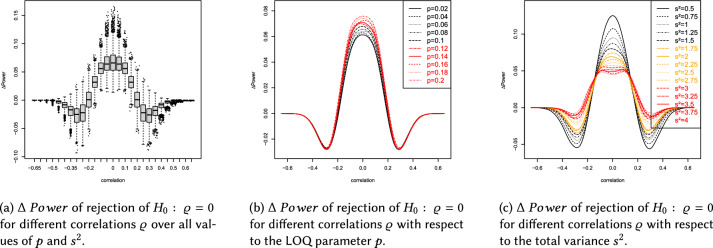
The data generating process is Cauchy

This section describes the results of the simulation study, which are summarized in Figs.  4b, 5, 6a and Tables 1, 2, 3, 4, 5.

**Fig. 7:**
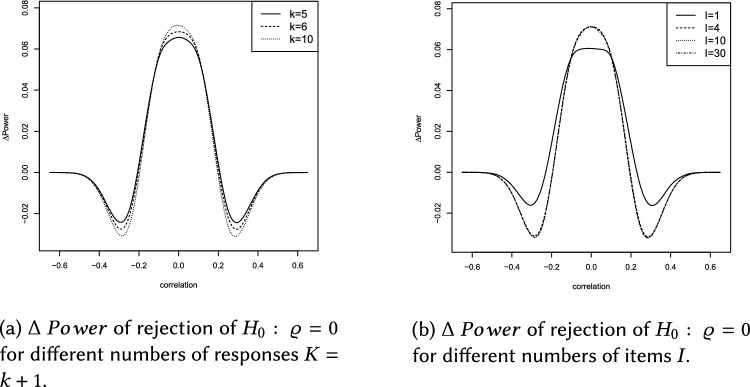
The data generating process is Cauchy

Below, we present the main results of the simulation with respect to the DGP, variances $s_{11},s_{22}\in \{0.25,$ $0.5,0.75,1,1.25,1.5,1.75,2\}$ (with total variance $s^2=s_{11}+s_{22}$) and values of $p\in \{0.02,0.04,\ldots ,0.2\}$ reflecting the LOQ. Please note that $\Delta \ Power>0$ indicates superiority of the ilr approach. Values of $\Delta \ Power$ were derived using the splinefun function of the R statistic software package, applying the fmm method of [51]. In brief the results can be summarized as follows:

1. The Laplace distribution yields $\Delta \ Power\in (-0.09,0.13)$, see Fig. 4a, and the Cauchy distribution yields $\Delta \ Power\in (-0.09,0.15)$, see Fig. 6a.
2. The influence of the LOQ parameter *p* on $\Delta \ Power$ seems to be independent of the DGP used during the simulation. Figures 4b, 5, 6b and Tables 2, 3 show that $\Delta \ Power$ increases as *p* increases.
3. Concerning a Laplace (Cauchy) DGP the ilr approach could cause a negligible (moderate) loss of statistical power if $0.2<\left| \varrho \right| <0.4$. Whereas, $\left| \varrho \right| \le 0.2$ yields $\Delta \ Power>0$ for arbitrary values of *p* (see Figs.  4b and  6b and Tables 2 and 3).
4. Concerning a Laplace (Cauchy) DGP and the total variance parameter $s^2$, $\Delta \ Power$ increases (decreases) as $s^2$ increases, compare Figs. 4c and 6c and Tables  4 and  5.
5. If the DGP is Cauchy increasing $s^2$ flattens the $\Delta \ Power$ curve (see Fig. 6c).
6. The total variance $s^2$ has a considerable effect on $\Delta \ Power$ (see Figs. 4c, 5, 6c). By contrast, the LOQ parameter *p* is less influential (see Figs. 4b, 5, 6b).
7. If $\left| \varrho \right| >0.4$ the ilr approach is neither superior nor inferior to the traditional evaluation because $\Delta \ Power\approx 0$.
8. Using the ilr approach a moderate (or sometimes large) increase of statistical power can be observed for the majority of sets of parameter combinations. It overcomes the marginal (or sometimes moderate) loss of statistical power compared to traditional correlation analyses.
9. The number of responses $K=k+1$ hardly affects $\Delta \ Power$, see Figs. 5a, 6, 7a.
10. A number of items $I\ge 4$ does not affect $\Delta \ Power$. However, a short LS consisting of $I=1$ item marginally affects $\Delta \ Power$, see Figs. 5b, 6, 7b.
11. Overall, the increase of $\Delta \ Power$ using a Laplace or Cauchy DGP seems comparable to the results of [8, 12, 14] assuming a normally distributed DGP and compliance with the CLT.

## Discussion and limitations

The Simplex affects correlation-based big data analytics. Evaluation of the ilr-transformed data instead of the raw data is expedient [15, 33, 34] and the results can be back-transformed by means of the inverse ilr transformation [11].

Consider the continuous bivariate Laplace distribution with center 0 and small variances. It is unimodal with a peak, has probability mass at the outer regions, and has less kurtosis than a normal distribution. Due to the heavy tails, a Laplace DGP is more likely to produce large absolute values compared to a normally distributed DGP. On the other hand, the small variance ensures that values close to 0 are very likely to be observed. If the variances are small it is expected that central random values in the Laplace distribution tend to be closer and have a smaller distance from the center 0. That is, values near the center are “denser” in the Laplace distribution than, e.g., in a normal distribution. Consider the relative distance *RD* of two neighboring responses.

10 $$\begin{aligned} RD=\frac{absolute \,distance \,of \,two \,responses}{range \,of \,the \,response \,scale} \end{aligned}$$

For two adjacent item responses (e.g. 2 and 3) we have $RD=1/4$. Consider the ilr transformation with $pLOQ=0.05$ yielding the ilr RS $\{-2.59, -0.73, 0, 0.73, 2.59\}$. The RD of the central values 0 and 0.73 is 0.73/5.18 = 0.14. The ilr transformed central item responses are thus closer together in absolute and relative terms than the untransformed responses. Obviously, the same effect can be observed for the discrete sets of corresponding possible means of ilr transformed and untransformed item responses. The example demonstrates that the ilr transformation moves central item response means closer together and elarges the distance of boundary values. Please note that the ilr approach provides more possible means of item responses than the traditional approach (see Sect. 3.4.2).

Assume a population correlation $\varrho$ close to 0. The set of possible means in the ilr space provides more values close to 0 than in the traditional data space (see Sect. 3.4.2). It is finer-grained. Thus, the sample correlation of the ilr transformed data tends to be a more precise estimate of the population correlation resulting in a larger statistical power of the correlation test.

A slight increase of the population correlation has less effect in the ilr transformed data space than in the traditional data space because the possible means are closer in the ilr space. That is, in the ilr space the sample correlation would remain almost unchanged while in the traditional data space it increases. Consequently, the sample correlation would underestimate the population correlation in the ilr space reducing the statistical power of the correlation test.

Further increasing the population correlation makes it easier for the correlation test to reveal that the null-hypothesis $\varrho =0$ is not true, irrespective of using traditional or ilr transformed data. Concerning the Cauchy distribution the same arguments are applicable. They explain the loss of statistical power for $0.2<\left| \varrho \right| <0.4$ if the DGP is heavy-tailed and the gain of statistical power if the DGP is normally distributed [8].

Partial correlations form a basic instrument in the analysis of big data sets consisting of large numbers of variables. Moreover, they contribute the regression coefficients in terms of multiple linear regression. The larger the number of variables is, the closer partial correlations or regression coefficients will be to 0 [52]. That is, assuming $0.05<|\varrho |<0.2$ seems plausible, making the potential losses in statistical power in the range $\left| \varrho \right| >0.2$ appear less important than the gains in the range $\left| \varrho \right| \le 0.2$.

The boxplots of Figs. 4a and  6a provide additional information. The trend of the medians is very similar to the trend of the splines. In the range $\left| \varrho \right| \le 0.2$, the heights of the boxes and the lengths of the whiskers are similar, meaning that the results are similarly reliable in that range. The height of the boxes is approximately 2 percentage points, indicating that the boxes represent a range of median ± 1%. Taken together, both pieces of information suggest a qualitatively adequate robustness of the results.

The values outside the whiskers indicate that there are scenarios that cause even more extreme changes in statistical power. In the range $\left| \varrho \right| \le 0.2$, there are more values above the upper whisker than below the lower whisker. This means that when extreme deviations occur, they tend to indicate an increase in statistical power induced by the ilr approach. In the range $\left| \varrho \right| >0.2$, the extreme deviations are more likely to be below the lower whisker, indicating a loss of power induced by the ilr approach. However, qualitatively, the range of extreme power increases in the range $\left| \varrho \right| \le 0.2$ is greater than the range of extreme power losses in the range $\left| \varrho \right| >0.2$. That is, the extreme increases are more pronounced than the extreme losses. This suggests that the ilr approach is generally superior to the traditional approach.

In the range $\left| \varrho \right| \ge 0.4$, the boxes narrow and the whiskers shorten, indicating an increasing robustness of the results. This is because the statistical power of the correlation test increases with increasing effect size. This increase occurs regardless of whether the data are analyzed traditionally or using the ilr approach. In both cases, the statistical power converges to 1 and therefore the difference converges to 0.

The gain in statistical power using the ilr approach is evident if the DGP is heavy-tailed (Laplace) or of infinite variance (Cauchy) and $0.05<|\varrho |<0.2$. The results are in coherence with [8, 12, 14] assuming a normally distributed DGP.

The increase of statistical power contributes to the problem of low statistical power (“underpowerment”), see [28–30]. Significances at the edge of non-significance must not be neglected in big data psychometric analyses. The ilr approach increases the statistical power and provides unbiased parameter estimates rendering psychometric profiles and characterizations of the target group more reliable. It is possible to decrease the sample size (i.e., the number of test individuals) while maintaining at least the same statistical power as in traditional data analysis reducing ethical issues [53] and increasing economic effort.

Overall, the results of the simulation study suggest that a breakdown of the CLT or the violation of the assumption of a normally distributed DGP hardly affects the ilr approach in correlation analyses.

In practice, any RS refers to a limited number of responses and applying the ilr approach also yields a limited ilr RS. Consequently, the underlying data generating process must have finite variance. However, as $p\rightarrow 0$ the range of the ilr RS approaches $\infty$. Thus, the underlying distribution could have large (and asymptotically infinite) variance. Therefore, the results of the simulation using the Cauchy distribution are asymptotically relevant in practice. Knowing that the ilr approach holds even for heavy-tailed distributions (Laplace) or distributions of infinite variance (Cauchy) is satisfying and provides additional confidence in big data analytics.

The negligible influence of the LOQ parameter *p* is in coherence with the findings of [8, 12, 14, 31, 32]. Concerning the properties of psychometric scales and the simulation results assuming $p=0.1$ seems plausible.

A limiting factor of the simulation is the finite number of scenarios. Many more practically relevant scenarios exist. However, it is impossible to account for every nuance (e.g., more or less heavy tailed distributions, symmetric vs. non-symmetric distributions, larger variances $s_{ii}$ ($i\in \{1,2\}$), different numbers of scale items $I\in \mathbb N$ or responses $k+1\in \mathbb N$, non-symmetric limits of quantification ($\delta _l$ and $\delta _u$) concerning the scale ends). Thus far, the results appear to be plausible and generalizable towards symmetric heavy-tailed distributions with common values of $I,\ k+1,\ s_{ii}$ and symmetric LOQ (i.e., $\left| \delta _l\right| =\left| \delta _u\right|$). However, further research on the influences of non-symmetric LOQ and non-symmetric data generating processes on $\Delta \ Power$ is necessary.

**Table 1:** Summary of $\Delta \ Power$ over all values of *p* and $s^2$

$\varrho$	Laplace	Cauchy
± 0.05	0.052	0.066
± 0.1	0.049	0.057
± 0.15	0.032	0.033
± 0.2	0.010	0.001
± 0.25	− 0.004	− 0.022
± 0.3	− 0.007	− 0.027
± 0.35	− 0.004	− 0.020
± 0.4	− 0.001	− 0.009

$\varrho$ denotes the true correlation

**Table 2:** Summary of $\Delta \ Power$ for different values of *p* over all values of $s^2$

$\varvec{\varrho }$	p				
	0.02	0.04	0.06	0.08	0.1
± 0.05	0.048	0.049	0.049	0.050	0.051
± 0.1	0.044	0.043	0.046	0.046	0.046
± 0.15	0.025	0.027	0.028	0.030	0.031
± 0.2	0.006	0.006	0.007	0.007	0.009
± 0.25	− 0.007	− 0.007	− 0.007	− 0.006	− 0.005
± 0.3	− 0.008	− 0.008	− 0.007	− 0.007	− 0.007
± 0.35	− 0.004	− 0.004	− 0.004	− 0.004	− 0.004
± 0.4	− 0.001	− 0.001	− 0.001	− 0.001	− 0.001

$\varvec{\varrho }$	p				
	0.12	0.14	0.16	0.18	0.2
± 0.05	0.053	0.053	0.054	0.055	0.057
± 0.1	0.049	0.052	0.052	0.055	0.056
± 0.15	0.033	0.033	0.037	0.039	0.041
± 0.2	0.010	0.011	0.013	0.016	0.018
± 0.25	− 0.004	− 0.003	− 0.003	− 0.002	0.000
± 0.3	− 0.007	− 0.006	− 0.006	− 0.006	− 0.005
± 0.35	− 0.004	− 0.003	− 0.003	− 0.003	− 0.003
± 0.4	− 0.001	− 0.001	− 0.001	− 0.001	− 0.001

$\varrho$ denotes the true correlation. The underlying data generating process is Laplace distributed

**Table 3:** Summary of $\Delta \ Power$ for different values of *p* over all values of $s^2$

$\varvec{\varrho }$	p				
	0.02	0.04	0.06	0.08	
± 0.05	0.059	0.060	0.063	0.063	0.065
± 0.1	0.048	0.050	0.052	0.053	0.056
± 0.15	0.025	0.027	0.028	0.029	0.032
± 0.2	− 0.003	− 0.003	− 0.002	− 0.002	0.000
± 0.25	− 0.024	− 0.024	− 0.023	− 0.023	− 0.023
± 0.3	− 0.027	− 0.028	− 0.027	− 0.028	− 0.028
± 0.35	− 0.019	− 0.020	− 0.019	− 0.020	− 0.019
± 0.4	− 0.009	− 0.009	− 0.009	− 0.009	− 0.010

$\varvec{\varrho }$	p				
	0.12	0.14	0.16	0.18	0.2
± 0.05	0.067	0.068	0.071	0.072	0.074
± 0.1	0.059	0.060	0.061	0.064	0.065
± 0.15	0.034	0.035	0.037	0.040	0.039
± 0.2	0.000	0.004	0.005	0.005	0.007
± 0.25	− 0.023	− 0.022	− 0.022	− 0.020	− 0.019
± 0.3	− 0.028	− 0.027	− 0.028	− 0.028	− 0.027
± 0.35	− 0.020	− 0.020	− 0.020	− 0.020	− 0.020
± 0.4	− 0.009	− 0.010	− 0.010	− 0.010	− 0.010

$\varrho$ denotes the true correlation. The underlying data generating process is Cauchy distributed

**Table 4:** Summary of $\Delta \ Power$ for different values of $s^2$ over all values of *p*

$\varvec{\varrho }$	$\varvec{s}^2$				
	0.5	0.75	1	1.25	1.5
± 0.05	0.035	0.042	0.049	0.050	0.053
± 0.1	0.026	0.032	0.036	0.041	0.046
± 0.15	0.010	0.013	0.019	0.021	0.025
± 0.2	− 0.006	− 0.007	− 0.002	0.001	0.003
± 0.25	− 0.009	− 0.014	− 0.013	− 0.011	− 0.009
± 0.3	− 0.008	− 0.009	− 0.011	− 0.009	− 0.010
± 0.35	− 0.003	− 0.005	− 0.005	− 0.004	− 0.005
± 0.4	− 0.001	− 0.001	− 0.002	− 0.001	− 0.001

$\varvec{\varrho }$	$\varvec{s}^2$				
	1.75	2	2.25	2.5	2.75
± 0.05	0.053	0.054	0.053	0.053	0.055
± 0.1	0.048	0.050	0.051	0.053	0.051
± 0.15	0.028	0.031	0.035	0.034	0.038
± 0.2	0.008	0.008	0.011	0.012	0.014
± 0.25	− 0.007	− 0.006	− 0.003	− 0.004	− 0.002
± 0.3	− 0.008	− 0.008	− 0.007	− 0.007	− 0.006
± 0.35	− 0.004	− 0.004	− 0.004	− 0.004	− 0.004
± 0.4	− 0.001	− 0.001	− 0.001	− 0.001	− 0.001

$\varvec{\varrho }$	$\varvec{s}^2$				
	3	3.25	3.5	3.75	4
± 0.05	0.054	0.053	0.053	0.051	0.050
± 0.1	0.055	0.053	0.056	0.057	0.055
± 0.15	0.040	0.040	0.043	0.046	0.047
± 0.2	0.017	0.018	0.023	0.026	0.024
± 0.25	0.000	0.002	0.003	0.005	0.008
± 0.3	− 0.006	− 0.003	− 0.003	− 0.002	0.000
± 0.35	− 0.003	− 0.003	− 0.002	− 0.003	− 0.002
± 0.4	− 0.001	− 0.001	− 0.001	− 0.001	− 0.001

$\varrho$ denotes the true correlation. The underlying data generating process is Laplace distributed

**Table 5:** Summary of $\Delta \ Power$ for different values of $s^2$ over all values of *p*

$\varvec{\varrho }$	$\varvec{s}^2$				
	0.5	0.75	1	1.25	1.5
± 0.05	0.114	0.099	0.089	0.081	0.076
± 0.1	0.079	0.073	0.067	0.063	0.062
± 0.15	0.033	0.031	0.030	0.029	0.028
± 0.2	− 0.015	− 0.015	− 0.012	− 0.010	− 0.007
± 0.25	− 0.047	− 0.043	− 0.039	− 0.036	− 0.033
± 0.3	− 0.056	− 0.049	− 0.043	− 0.041	− 0.036
± 0.35	− 0.043	− 0.037	− 0.032	− 0.028	− 0.025
± 0.4	− 0.026	− 0.020	− 0.016	− 0.014	− 0.013

$\varvec{\varrho }$	${}^2s$				
	1.75	2	2.25	2.5	2.75
± 0.05	0.071	0.066	0.063	0.060	0.057
± 0.1	0.057	0.057	0.054	0.054	0.053
± 0.15	0.029	0.030	0.030	0.034	0.035
± 0.2	− 0.005	− 0.003	− 0.003	0.003	0.008
± 0.25	− 0.030	− 0.027	− 0.025	− 0.017	− 0.014
± 0.3	− 0.035	− 0.031	− 0.029	− 0.022	− 0.020
± 0.35	− 0.024	− 0.021	− 0.020	− 0.016	− 0.013
± 0.4	− 0.011	− 0.010	− 0.010	− 0.007	− 0.006

$\varvec{\varrho }$	$\varvec{s}^2$				
	3	3.25	3.5	3.75	4
± 0.05	0.055	0.052	0.050	0.048	0.047
± 0.1	0.053	0.051	0.050	0.049	0.049
± 0.15	0.037	0.039	0.038	0.037	0.040
± 0.2	0.010	0.014	0.017	0.018	0.020
± 0.25	− 0.010	− 0.008	− 0.004	− 0.001	0.000
± 0.3	− 0.016	− 0.013	− 0.012	− 0.011	− 0.007
± 0.35	− 0.011	− 0.010	− 0.009	− 0.007	− 0.007
± 0.4	− 0.005	− 0.004	− 0.004	− 0.003	− 0.003

$\varrho$ denotes the true correlation. The underlying data generating process is Cauchy distributed

## Data Availability

There are no real data available. The authors agree to share the R codes used in the simulation process upon request.
